# Danshen enhanced the estrogenic effects of Qing E formula in ovariectomized rats

**DOI:** 10.1186/s12906-016-1146-5

**Published:** 2016-06-23

**Authors:** Jian-mei Zhang, Jin Li, Er-wei Liu, Hong Wang, Guan-wei Fan, Yue-fei Wang, Yan Zhu, Shang-wei Ma, Xiu-mei Gao

**Affiliations:** School of Traditional Chinese Medicine, Tianjin University of Traditional Chinese Medicine, Tianjin, 300193 People’s Republic of China; Institute of Traditional Chinese Medicine, Tianjin University of Traditional Chinese Medicine, 312 Anshan xi Road, Tianjin, 300193 People’s Republic of China

**Keywords:** *Danshen*, Estrogenic effects, ERα, ERβ, Menopause, *Qing E formulaplus Danshen*, Rats

## Abstract

**Background:**

Menopause is characterized by a decrease in life quality due to the appearance of uncomfortable symptoms. Nowadays, Understanding menopause-associated pathophysiology and developing new strategies to improve the treatment of menopausal-associated symptoms is an important issue. Our study was to evaluate the synergistic effects of *Danshen* (*salvia miltiorrhiza bunge*) and the phytoestrogenic effects of 3 *modified Qing E* formulas, to explore a better formula for menopausal disorders.

**Methods:**

100 rats were randomized into 5 groups: Sham (Sham operation group), OVX (model group of ovariectomized rat), BDL (group with low concentration of *Qing E Formula*), BDH (group with high concentration of *Qing E Formula*) and BDD (group with high concentration of *Qing E Formula Plus Danshen*), receiving vehicle and extract of different modified *Qing E formula* respectively. The food intake, body weight, uterus weight, blood levels of triglycerides (TG), total cholesterol (TC) and cholesterol fractions were assessed. The mammary glands and uterus were morphologically analyzed. The bone density of tibias were measured by peripheral quantitative computed tomography (pQCT). Additionally, luciferase induction assays were performed in Hela cells with the mixtures derived from *Qing E formula plus Danshen* (BDD).

**Results:**

*Qing E formula plus Danshen* significantly increased the uterus wet weight, enhanced the thickness of uterine wall, endometrial epithelium and glandular epithelium, improved trabecular bone and total density evidently, reduced the levels of low density lipoprotein cholesterol (LDL-C) and TG, possessed notable estrogen receptor beta (ERβ) and estrogen receptor alpha (ERα) agonist activity.

**Conclusion:**

*Qing E formula plus Danshen* exerted more evident estrogen-like effects, thus it has a potential therapeutic use to treat menopausal disorders.

## Background

Menopause is characterized by an altered hormonal status and by a decrease in life quality due to the appearance of uncomfortable symptoms. Nowadays, with increasing life span, women spend one-third of their lifetime under menopause. Understanding menopause-associated pathophysiology and developing new strategies to improve the treatment of menopausal-associated symptoms is an important topic in clinics [1]. Hormone-replacement therapy (HRT) or estrogen-replacement therapy (ERT) is recommended for postmenopausal women primarily for reduction of menopausal symptoms and prevention of osteoporosis and cardiovascular disease. However, the treatment with estrogens after menopause may include a potential increase in the risk of breast cancer (relative risk seems to be about 1.3 after at least 8 years of HRT) [2], an increase in vaginal bleeding and an increased risk of endometrial cancer [3, 4]. Because of this, there is increasing interest in the use of plant-derived estrogens, also known as phytoestrogens.

Phytoestrogen is present in a wide variety of plant products, especially tonic and blood-activating Chinese herbs, with many categories of ingredients including lignans, isoflavonoids, coumestans and resorcyclic acid lactones, all of which bind estrogen receptors, but a lower binding affinity than steroidal estrogens. Phytoestrogen exerts estrogenic and/or anti-estrogenic effects inherently or after being conversed by intestinal flora against menopausal symptoms and a variety of disorders, including hot flushes, cardiovascular disease, cancer, hyperlipidemia, osteoporosis, and various forms of chronic renal disease, without serious side effects [5].

In China, *Qing E Wan* is one of the most well-known Herbal formulas, which was published in “Taiping Hui Min He Ji Ju Fang” in Song Dynasty (about year 1148), and composed of 4 kinds of herbs: *Duzhong* (*eucommia bark*), *Buguzhi* (*psoralea corylifolia*), *walnut* meat and *garlic. Qing E Wan* is also used medicinally in Pharmacopoeia of the People's Republic of China (year 2005) to relieve back pain, increase stamina, make bones and muscles 'strong' and to hasten recovery from fatigue, which are female hormone-related pharmacological effects. But all the herbs in *Qing E Wan* are hot-natured that is only suitable for kidney-yang deficiency (cold syndrome) patients according to Traditional Chinese Medicine (TCM).

In our research, only the two main herbs, *Duzhong* and *Buguzhi*, of *Qing E Wan* were left, and named as Qing E Fomula (BDL & BDH groups). For improving *Qing E Wan* to be more suitable for general patients (including heat syndrome) and the pathogenesis of menopause—kidney deficiency and blood stasis [6], a traditional Chinese herbal medicine, *Danshen*, was added into another research group named as *Qing E formula plus Danshen* (BDD).

The present study was conducted in order to evaluate the synergistic effects of *Danshen* and the estrogenic effects of 3 modified *Qing E formulas*, by oral gavages in the ovariectomized rats models of menopause and luciferase induction assays in Hela cells, to explore a better formula for the menopausal treatment.

## Methods

### Experimental animals

Postpartum female Sprague–Dawley rats (6-month-old, weighting 270士50g) were purchased from Shanchuanhong laboratory animals technology co. ltd, Tianjin, China (license No. SCXk 2009–0001). All rats were housed at the Experimental Animal Center, Tianjin University of Traditional Chinese Medicine (TJUTCM), on sawdust in the plastic bottomed cages at 22–24 °C under a 12 h light–dark cycle, and were provided with rodent chow and tap water ad libitum. All procedures were approved by the Animal Care and Use Committee of TJUTCM and conform to the Guide for the Care and Use of Laboratory Animals published by the U.S. National Institutes of Health (NIH Publication number 85–23, revised 1996).

### Herbs

The herbs of *Buguzhi*, *Duzhong* and *Danshen* were provided by Institute of Traditional Chinese Medicine, TJUTCM. Drug lot numbers were: 20090620. The extracts of *Buguzhi*, *Duzhong* and *Danshen* were prepared by pharmacist Liu and Wang in the above institute. The extraction rates of *Buguzhi*, *Duzhong* and *Danshen* are 20, 12 and 52.6 % respectively. The essential components of *Buguzhi* are bakuchiol and flavones; *Duzhong*’s are flavones, iridoid and lignans; *Danshen*’s are tanshinones and salvianolic acids. The dried herbal extractions were immersed into boiling water with 10 ml of 0.3 % carboxymethylcellulose sodium solution for 30 min each group. Then the water solution was stored at 4 °C for administration.

### Grouping of animals and herbal administration

One hundred rats were verified at normal estrus stage with vaginal smear and Papanicolaou (PAP) staining [7] after 10 days’ acclimation. Then all were anesthetized with chloral hydrate (3 ml/kg, TCI, China). Of these animals, 20 underwent a sham operation (sham group), while 80 were bilaterally ovariectomized (OVX). Two weeks later, the OVX rats were randomized into four groups of twenty animals each (OVX group, BDL group, BDH group and BDD group). The sham and OVX model groups received vehicle (0.3 % of carboxymethylcellulose sodium solution and purified water). The BDL and BDH groups received low- and high-dose extract of modified *Qing E formula* at the human equivalent dose of 250 and 500 mg/kg/day, respectively. The BDD group received high-dose extract of *Qing E formula plus Danshen* at dose of 1815 mg/kg/day. The details are as follows:

In humans, the clinical dosages of *Buguzhi*, *Duzhong* and *Danshen* are respectively about 4 g/day, 10 g/day and 10 g/day of dried herb recorded in the Chinese Medicine textbook. Considering the average body weight of an adult as 60 kg, to convert the human dose to rat dose equivalent, the amounts would be 0.42 g/kg/day, 1.05 g/kg/day and 1.05 g/kg/day (4 g/day/60 kg × 6.3, 10 g/day/60 kg × 6.3 and 10 g/day/60 kg × 6.3,) according to van Miert [8]. The total dosage of *Buguzhi* and *Duzhong* was 1.47 g/kg/day (0.42 + 1.05) for rats. Since very notable uterotrophic effect wasn’t shown up by using 1.47 g/kg/day of *Buguzhi* and *Duzhong* in the pre-test. So the herbs was lightly increased to 1.75 g/kg/day (0.5 g/kg/day of Buguzhi and 1.25 g/kg/day of *Duzhong*) as group BDL in this research. The BDH was 3.5 g/kg/day (2 times of BDL); BDD was 6 g/kg/day (2 times of BDL plus *Danshen*, the amount of *Danshen* was equal to *Duzhong*). The extract yields of *Buguzhi*, *Duzhong* and *Danshen* were 20.0, 12.0 and 52.6 % of the raw material respectively. Therefore, the extract amounts of BDL, BDH and BDD were 0.25 g/kg/day (0.5 g/kg/day × 20 % + 1.25 g/kg/day × 12 %), 0.5 g/kg/day (2 times of BDL) and 1.815 g/kg/day (2 times of BDL plus *Danshen* = 0.5 g/kg/day +2.5 g/kg/day × 52.6 %).

Half of animals were administrated for 6 weeks, the other half for 12 weeks by oral gavages in a volume of 10 ml/kg. Their body weight and food intake were weekly recorded throughout the whole experiment period.

### Serum and organ collection

After 6 weeks of treatment, half of animals were subjected to overnight fasting, then weighed and sacrificed under chloral hydrate (3 ml/kg, TCI, China) anesthesia between 8:00 and 12:00. Their blood was collected from the abdominal aorta into polypropylene tubes and kept at 4 °C for 2–4 h. The serum was obtained after centrifugation of these blood samples at 3000 rpm for 15 min at a temperature of 4 °C, then aliquoted in quadruplets into 2 ml polypropylene tubes and stored at −20 °C until further analysis.

The fifth mammary glands were collected and stored in 10 % buffered formalin for histopathologic examination and assessment of epithelial proliferation. The abdominal cavity was opened with a longitudinal cut and the uteri were removed. These uterine horns were dissected free of adhering fat and mesentery. After weighing, one uterine horn was fixed in 10 % buffered formalin for histological evaluation; The contralateral horn was transferred into 2 ml polypropylene tubes, frozen in liquid nitrogen, then stored at −80 °C for further analysis.

Twelve weeks later, the rest of rats were sacrificed under chloral hydrate (3 ml/kg, TCI, China) anesthesia. Their right tibias were dissected out, cleaned away all the soft tissues, placed in 70 % ethanol and stored at 4 °C for scanning by pQCT.

### Blood lipid test

The TG, TC, serum high density lipoprotein cholesterol (HDL-C), and LDL-C levels were measured by standard colorimetric methods using a semi-automatic biochemical analyzer type Microlab 300 (Vital Scientific, Netherlands). The kits were purchased from Zhongsheng Beikong Bio-technology and Science Inc. (Beijing, China).

### Gonadal hormone test

The rest of serum samples were taken to the Clinical Laboratory of Tianjin Medical University General Hospital, where the serum 17β-estradiol, follicle-stimulating hormone (FSH) and luteinizing hormone (LH) were determined using the radioimmunoassay kit supplied by DSL co., Czech.

### Mammary gland and uterine histology

All the formalin-fixed mammary glands and uterine horns were embedded in paraffin, cut into 4-mm thick slices, and stained with hematoxylin-eosin (H&E) for morphological analysis. The histological structure changes were observed under a light microscope, including the morphology and polarity of epitheliums, the thickness of uterine wall and endomembrane, the height of glandular epithelium and endometrium epithelium. Then, the color photographs were collected under a medical digital image acquisition system (Soft Imaging System, Munster, Germany).

### Estrogenic activities test

To investigate the estrogenic activities of the mixtures derived from *Qing E formula plus Danshen* (BDD), luciferase induction assays were performed in Hela cells. Hela cells were cultured as described previously [9].

Mammalian expression vectors, ERαand ERβ, were gifts from Dr. Karas (Tufts Medical Center, Boston, USA). The luciferase reporter plasmid, carrying 3 × vitellogenin ERE (estrogen response element, ERE), was kindly provided by Dr. J. Zhang (Nankai University, Tianjin, China). Cells were plated, in triplicate, in 24- well plates at a density of 2 × 10^5^ cells/well in 10 % CD-FBS. After attachment and growth for 24 h, the cells were cotransfected with the reporter plasmid ERE-TK-Luc and ERα/β expression plasmids. pRL-TK plasmid, which contains a Renilla luciferase gene, was used as a control for normalising transfection efficiency. Transfection was carried out for 18 h in serum-free, antibiotic-free DMEM media, using Lipofectamine 2000 (Invitrogen/Life Technologies, Carlsbad, CA) according to the manufacturer’s instructions; medium was removed, Hela cells were treated with various drug concentrations of aqueous extract from *Qing E formula plus Danshen* (0.1, 1, 10, 100 μg/ml, respectively), and incubated for 24 h following transfection. E2 (10 nM) was used as positive control. The cells from each well were lysed. Aliquots from each well were divided into two 96-well plates for luciferase and Renilla activity determination using a Topcount NXT luminescence counter (Packard Instrument Company, Meriden, CT). Experiments were performed at least three times and the data were assessed as units of firefly luciferase activities normalised to the Renilla luciferase control activities from individual wells.

### pQCT measurements

All the ethanol-fixed tibias were posted to Shanghai Institute of Materia Medica,Chinese Academy of Sciences,for BMD analysis using a pQCT apparatus (Stratec XCT Research SA, Stratec Medizintechnik GmbH, Pforzheim, Germany). The modalities of proximal tibias were display by vertical scanning. Then the tibias of epiphyseal line down to 3.0 mm (containing a high percentage of trabecular structure) and 12.0 mm (mainly cortical structure) were faulting scanned using a 0.46-mm collimation (4 × 10^5^counts/s) and a 0.08-mm voxel size. Thresholds for segmentation of trabecular and cortical bone were set at 300 mg/cm3 and 900 mg/cm3, respectively.

### Statistical analysis

All statistical analysis was performed by using Statistical package for the Social Sciences (version 16.0; SPSS, Inc., Chicago, IL, USA). The analysis of variance (ANOVA), Post-hoc Bonferroni test, and paired t test were used to evaluate the effects of each variable and to reveal the statistical significance. *P* value <0.05 was considered significant.

## Results

### Effects on the food intake, body weight and uterus weight

Rats in all experimental groups had similar initial body weights and similar food intake. All the body weights increased continuously during the research. In the OVX group, the body weight gains showed notable increase (Table 1), and BDH group was significant increase also. As expected, ovariectomy induced obvious uterus atrophy and the decreased uterus index. But the uterus wet weight in BDD group showed significant increasecompared with the OVX group.

**Table 1:** Effects on the food intake, body weight and uterus weight

Groups	Food intake per average week (g)	Initial body weight (g)	Final body weight (g)	Body weight gain (g)	Uterine wet weight (g)	Uterine weight/body weight ratio
Sham (*n* = 9)	30.01 ± 5.61	265.11 ± 31.07	284.67 ± 43.85	19.56 ± 4.83	0.566 ± 0.13	0.2124 ± 0.05
OVX (*n* = 8)	30.60 ± 5.63	255.75 ± 35.03	302.25 ± 49.40	46.50 ± 7.08**	0.176 ± 0.03**	0.0641 ± 0.022**
BDL (*n* = 9)	27.83 ± 6.26	253.11 ± 25.95	288.11 ± 34.52	35.00 ± 8.77	0.193 ± 0.03**	0.0720 ± 0.02**
BDH (*n* = 9)	27.82 ± 3.24	245.56 ± 24.96	282.67 ± 29.21	38.22 ± 5.10*	0.204 ± 0.03**	0.0776 ± 0.01**
BDD (*n* = 9)	28.80 ± 6.06	260.63 ± 53.64	289.13 ± 50.76	28.50 ± 4.63	0.211 ± 0.03**^▲^	0.078 ± 0.01**

Data are expressed as mean ± S.E.M. Means in columns with superscript are significantly different. *P < 0.05,**P < 0.01 *vs*. Sham group; ^▲^ *P* < 0.05 *vs*. OVX

### Effects on the blood lipid

Table 2 shows a significant increase in serum TC, LDL-C, HDL-C and TG in OVX group compared with sham group. Administrating the 3 formulas to OVX rats resulted in a significant reduction in the LDL-C, and TG levels compared with OVX rats. In terms of serum TC, no statistically significant difference was seen among the three treated groups. Although HDL-C levels of all the OVX rats were still higher than the sham group’s, HDL-C/TC were significantly increased in BDH and BDD group.

**Table 2:** Effects on the blood lipid

Groups	TG (mmol/l)	TC (mmol/l)	HDL-C (mmol/l)	LDL-C (mmol/l)	HDL-C/TC (%)
Sham (*n* = 9)	0.772 ± 0.10	2.406 ± 0.18	0.884 ± 0.05	0.414 ± 0.05	37.23 ± 3.48
OVX (*n* = 8)	1.644 ± 0.31**	2.931 ± 0.22*	1.046 ± 0.04**	0.676 ± 0.13**	36.34 ± 4.01
BDL (*n* = 9)	1.006 ± 0.15^▲▲^**	2.824 ± 0.18	1.078 ± 0.05**	0.396 ± 0.06^▲▲^	38.46 ± 2.44
BDH (*n* = 9)	0.666 ± 0.03^▲▲^	2.682 ± 0.06	1.131 ± 0.03**	0.411 ± 0.04^▲▲^	42.18 ± 2.19^▲▲*●^
BDD (*n* = 9)	0.478 ± 0.02^▲▲●^	2.757 ± 0.10	1.095 ± 0.02**	0.399 ± 0.02^▲▲^	39.93 ± 2.67^▲^

Data are expressed as mean_S.E.M. Means in columns with superscript are significantly different. *P < 0.05,**P < 0.01*vs*. Sham group; ^▲^ *P* < 0.05,^▲▲^ *P* < 0.01 *vs*. OVX group; ^●^ *P* < 0.05 *vs*. BDL. *TG* triglyceride, *TC* total cholesterol, *HDL*-*C* high density lipoprotein cholesferol, *LDL*-*C* low density lipoprotein cholesferol

### Effects on the gonadal hormones

Ovariectomied rats showed significant increase in FSH and LH, but notable decrease in E_2_ (Table 3). After administration of the 3 formulas, E_2_ was mildly increased in each treated group. As for FSH and LH, no statistically significant difference was observed.

**Table 3:** Effects on the gonadal hormones

Groups	FSH (ng/ml)	LH (ng/ml)	E_2_ (pg/ml)
Sham (*n* = 9)	5.86 ± 0.77	0.38 ± 0.03	14.05 ± 1.09
OVX (*n* = 8)	51.40 ± 5.82**	4.22 ± 0.36**	8.26 ± 0.76**
BDL (*n* = 9)	45.81 ± 4.20**	4.64 ± 0.61**	10.40 ± 0.98*
BDH (*n* = 9)	53.01 ± 5.25**	3.81 ± 0.34**	10.83 ± 1.46*
BDD (*n* = 9)	51.11 ± 4.10**	4.71 ± 0.38**	10.87 ± 0.87*

Data are expressed as mean_S.E.M. Means in columns with superscript are significantly different. *P < 0.05,**P < 0.01*vs*. Sham group; *FSH* follicle-stimulating hormone, *LH* luteinizing hormone, *E*_*2*_ estradiol

### Effects on the morphology of mammary and uterine tissue

**Fig. 1:**
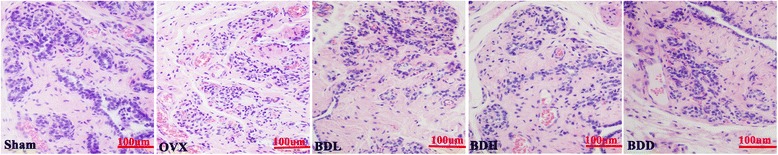
Effects on the morphology of mammary. The longitudinal section of the mammary in each group. After the administration of 3 modified *Qing E formulas*, the mammary glands showed different degrees of slighter atrophy in comparison with the OVX group

**Fig. 2:**
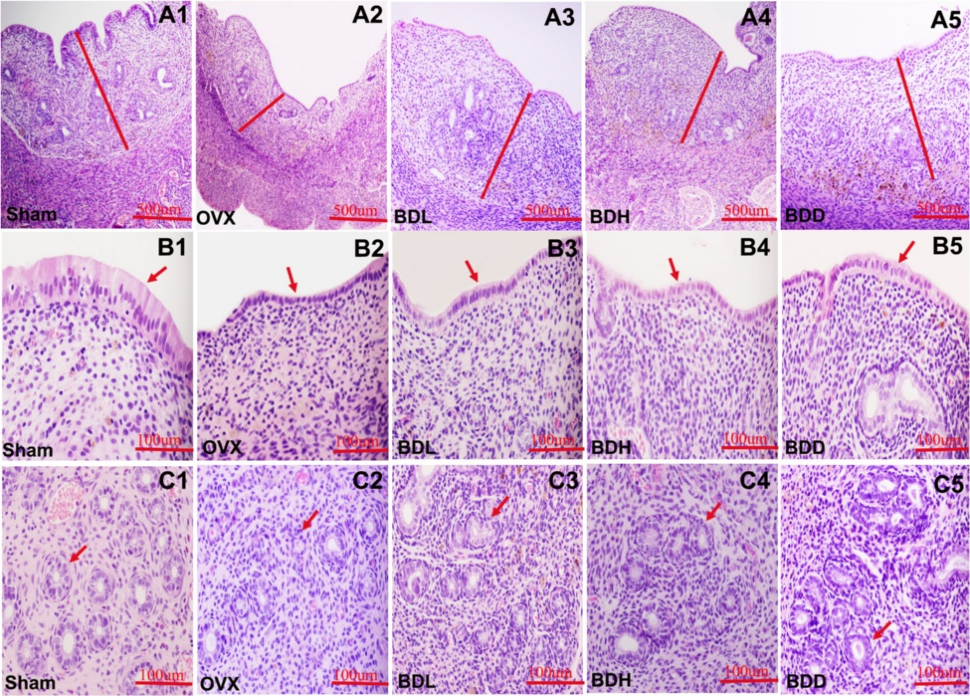
Effects on the morphology of uterine tissue. **A1**-**A5** showed the transverse section of the uterus in each group. The endometrium thickness of all the ovariectomized rats was significantly decreased compared with the sham group, but mildly increased by all the formulas. Redline - the endometrium. (100×). **B1**-**B5** showed transverse section of the uterus in each group. The endometrium epithelium thickness of all the ovariectomized rats was significantly decreased compared with the sham group, But significantly increased by *Qing E formula plus Danshen*. Arrow- epithelium. (400×). **C1**-**C5** showed transverse section of the uterus in each group. The uterine glandular epithelium thickness of all the ovariectomized rats was significantly decreased compared with the sham group, But significantly increased by all the formulas. Arrow-uterine gland. (400×)

Figure 1 shows the microscopic preparations of representative mammary glands from one animal per treatment group. In the sham group, the structure of mammary glands were normal. The alveoli and ducts of mammary gland were simple cuboidal epithelium or columnar epithelium with larger cell size, round or oval nuclei. In the OVX group, the mammary glands were severely atrophied. The alveoli and ducts of mammary gland were made up of low-cube-shaped epithelium with smaller cell size and shrunk nuclei. However, in all 3 treatment groups, the mammary glands showed different degrees of slighter atrophy in comparison with the OVX group. But the alveoli and ducts of mammary gland were still simple cuboidal epithelium. Figure 2 shows microscopic preparations of representative uteri from one animal in each treatment group. The quantitative data obtained from uterine tissues of all animals are shown in Table 4. Every uterus tissue morphology in the sham group was normal. But in the OVX, uterus atrophy was observed in all structures. The endometrium was composed of inactive cuboidal cells, and the connective tissue was an unorganized lax syncytium with contracted nuclei. The quantity of endometrial glands in subintima was decreased. Simultaneously, the size and glandular cavity of glands were shrunken. While slight atrophy of uterus was observed in all formula treated groups. The glandular epithelium thickness showed obvious difference at various degrees in all formula treatment groups in comparison with the OVX. Additionally, and the thickness of uterine wall and endometrial epithelium also showed significant increase in the BDD group compared with the OVX.

**Table 4:** Uterine tissue morphology

Groups	Uterine wall thickness (μm)	Endometrium thickness (μm)	Endometrial epithelial thickness (μm)	Glandular epithelium thickness (μm)
Sham (*n* = 9)	1397.2 ± 232.7	492.2 ± 214.66	31.15 ± 9.62	59.18 ± 14.03
OVX (*n* = 8)	732.9 ± 158.9**	289.7 ± 86.9**	25.16 ± 5.04*	31.44 ± 6.59**
BDL (*n* = 9)	750.3 ± 171.5**	337.9 ± 108.9**	24.44 ± 4.25*	45.97 ± 10.94*^▲^
BDH (*n* = 9)	774.6 ± 210.5**	321.4 ± 87.3**	24.20 ± 2.24*	47.92 ± 11.75*^▲▲^
BDD (*n* = 9)	907.4 ± 71.2**^▲^	343.3 ± 68.53**	31.03 ± 5.55^▲^	43.57 ± 8.88**^▲^

*P < 0.05,**P < 0.01*vs*. Sham group; ^▲^ *P* < 0.05,^▲▲^ *P* < 0.01 *vs*. OVX group

### Effects on the BMD

**Fig. 3:**
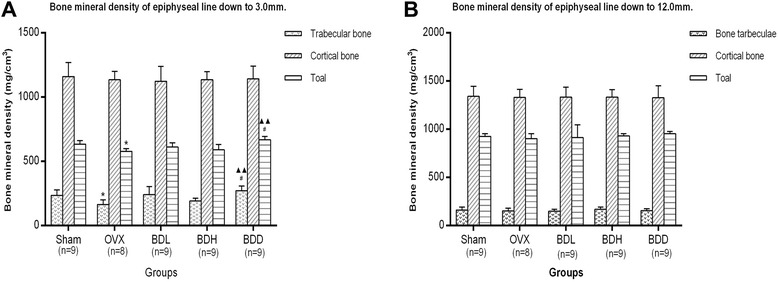
Effects on the BMD. **a** showed that the density of trabecular bone and total bone density were significantly improved by *Qing E formula Plus Danshen* at epiphyseal line down to 3.0 mm; **b** showed that there was no apparent difference shown at the epiphyseal line down to 12.0 mm. ^★^ *P* < 0.05 *vs*. Sham group; ^▲▲^ *P* < 0.01 *vs*. OVX group; ^#^ *P* < 0.05 *vs*. BDH group

**Fig. 4:**
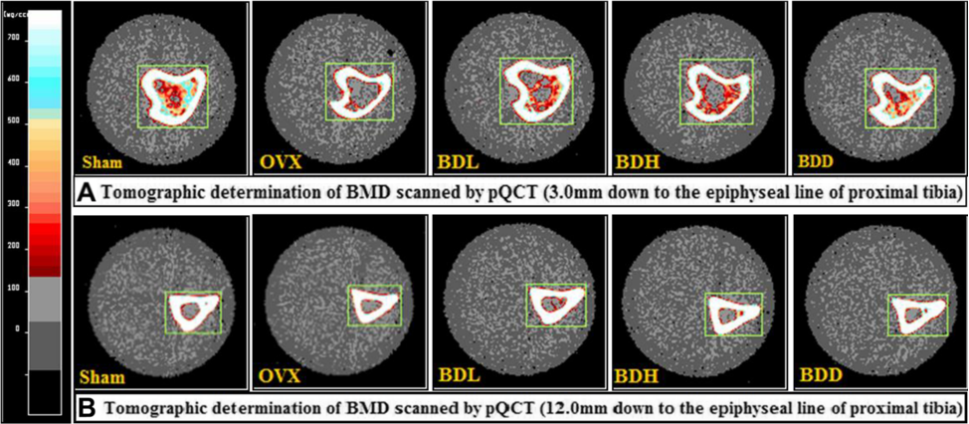
Tomographic determination of BMD scanned by pQCT (3.0 mm & 12.0 mm down to the epiphyseal line of proximal tibia). The scale bar of BMD is from 0 to over 1000 shown at the left with color variation from black, gray, red, blue,…to white. In Fig. **4** **-** **a**, there was evidently higher intensity component with red and blue signal values in the cancellous bone area in group BDD comparing to OVX, which was mostly in gray; however, the BMD of cortical bone didn’t show any notable difference in fig. **4-b**.

From the data and images of cancellous bone in the proximal tibial metaphysis (the epiphyseal line down to 3.0 mm) scanned by pQCT (Figs. 3 and 4), it was clearly seen that ovariectomy resulted in the reduction of trabecular bone and total density. However, those were significantly improved by *Qing E formula Plus Danshen*. But no apparent difference was observed at the epiphyseal line down to 12.0 mm.

### Effects on the expression of estrogenic activities

**Fig. 5:**
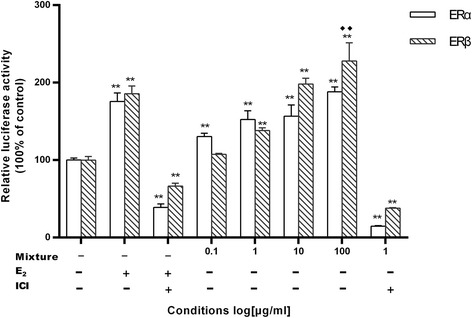
Effects on the expression of estrogenic activities. Transcription of ERα and ERβ activated by the mixture of BDD in Hela cells. Hela cells were transiently cotransfected with pERE-luc, pRL-TK, and ERα or ERβ by lipofection. The cells received no treatment, treatment with 10 nM E_2_, or treatment with varying mass concentrations of each of the mixture alone. Estrogen-stimulated luciferase activity of ERs was measured and the data was presented as the fold-increase over control + standard deviation, and fold-increase representative of three experiments done in triplicate. ** *P* < 0.01 vs. control; ^◆◆^ *P* < 0.01 vs.E_2_

E_2_ and the mixtures from *Qing E formula plus Danshen* (BDD) did increase luciferase activity for ERs. The mixtures at varying mass concentrations (0.1, 1, 10, 100 μg/ml, respectively) all caused a significant increase of luciferase activity in ERα, while at these concentrations (1, 10, 100 μg/ml, respectively) caused a significant increase of luciferase activity in ERβ, both in a dose-dependent manner. When the potencies were compared, it was evident that none of the mixtures was more potent than E_2_ in ERα, but the mixture at the concentration of 100 μg/ml showed noticeably more potent than E_2_ in ERβ. When the cells were simultaneously treated with the test mixture and a pure ER antagonist, ICI 182,780, at a concentration of 1 μg/ml, luciferase activity was greatly suppressed. The test mixtures transactivated better through ERβ than ERα (Fig. 5).

## Discussion

Menopause leads to a wide range of symptoms and disorders, including hot flashes, night sweats, sleeping problems, emotional dysfunction, endocrine disorders including hyperlipidemia, hypertension, hyperglycemia, and osteoporosis etc.. Phytoestrogens have been applied for compensation of hormone deficiency in the menopause [10]. Phytoestrogens are present in certain edible plants being most abundant in soy; they are structurally and functionally analogous to the estrogens. Among them, isoflavones and coumestans are the most extensively studied groups. Isoflavones are present in different edible plants being most abundant in soy [11–13]. However, soy is known as allergenic food at least for some populations [12, 14]. In regard to osteoporosis, the latest review concluded that ‘evidence points to a lack of a protective role of soy isoflavones in the prevention of postmenopausal bone loss’ [15]; Dewell’s findings suggested that soy-derived phytoestrogens did not significantly alter serum lipoproteins in postmenopausal women and, therefore, might not effectively reduce the risk of Coronary Artery Disease [16]. As for other phytoestrogens, in animals, the intake of clover was reported to impact fertility and morphogenesis of ovaries in sheep [17]. Therefore, we are still on the way to seek more effective phytoestrogens for menopausal treatment.

According to the TCM theory, the pathogenesis of menopause is kidney deficiency and blood stasis. Kidney deficiency is the fundamental pathogenesis. Therefore, we kept looking for the most proper formula specialized in nourishing kidney and promoting blood circulation. *Qing E Wan* has been already used as an important formula for treating postmenopausal osteoporosis in Chinese traditional medicine. For improving it to be more suitable for menopausal treatment, *Danshen* was added in a research group. *Qing E formula plus Danshen* is composed of *Duzhong*, *Buguzhi* and *Danshen*, which are all well-known traditional Chinese medicine and applied in clinic for thousands years. In this formula, *Duzhong* and *Buguzhi* are the principal herbs. *Duzhong* is the bark of the Chinese medicinal herb, *Eucommia ulmoides Oliv*., warm in nature, playing an important role in nourishing the liver and kidney, strengthening bone and muscle, and preventing abortion etc. [18–20]. *Buguzhi* is the seed of *psoralea corylifolia L*., warm in nature, efficacious in warming kidney yang and antidiarrheal. It has been applied as a tonic or an aphrodisiac agent and commonly used as a remedy for bone fracture, osteomalacia and osteoporosis in China [21]. A in vitro research also demonstrated that *Buguzhi* exhibited osteoblastic proliferation stimulating activity in UMR106 cell line cultured, and might stimulate bone formation or have potential activity against osteoporosis [22]. Danshen is the root of Salvia miltiorrhiza bunge, considered to have an action of quickening the blood and dispelling stasis, and is frequently used to treat related disorders of blood stasis such as cerebrovascular accident and ischemic heart disease [23]. It has been reported to have an anti- platelet aggregation effect [24], and support bone healing [25]. Additionally, *Danshen*, *Buguzhi*, *Duzhong* and *Danshen* were also confirmed to possess estrogenic activity respectively [26–28]. Therefore, we assumed that *Danshen* could enhance the estrogenic effects of *Qing E formula*.

Studies on rodent and non-human primates rely on an ovariectomized model of surgical menopause, resulting in abrupt withdrawal of estrogen. The uterus is one of the major target tissues of endogenous and exogenous estrogens [29]. Our study demonstrated that ovariectomized rats showed a significant decrease in serum estrogen concentration (Table 3), uterine wet weight (Table 1) and endometrial thickness (Table 4) compared with sham-operated rats. The reduction in endometrial thickness was caused by the lack of estrogen secreted by the ovaries. Administration of three *Qing E formulas* to ovariectomized rats for 6 weeks slightly increased serum estrogen concentration, uterine wet weight and endometrial thickness. However, *Qing E formula plus Danshen* increased the Uterine wet weight significantly (Table 1) compared with low dose and high dose of *Qing E formula*., which was due to the notably increased thickness of uterine wall, endometrial epithelium and glandular epithelium (Table 4; Fig. 2). But no obvious overstimulation was observed in the morphology of mammary and uterine tissue (Figs. 1 and 2). Therefore, *Danshen* enhanced the estrogenic effects of *Qing E formula*. In another word, *Qing E formula plus Danshen* could be considered a safe and more effective complementary or alternative treatment for menopausal syndrome.

For identifying the estrogenic activities of *Qing E plus Danshen*, we assessed the effects of *Qing E plus Danshen* on ERα alone and ERβ alone in HeLa cells after in vitro research. It has been proposed that tissue-specific estrogenic and/or antiestrogenic actions of certain xenoestrogens may be associated with alterations in the tertiary structure of ERα and/or ERβ following ligand binding. ERα is the predominant ER found in uterus and liver, whereas ERβ is highly expressed and is almost the exclusive ER in ovarian granulosa cells [30]. The presence of ERα is associated with the proliferative effects of estrogens, whereas the bulk of current evidence implies that ERβ as growth suppressive properties [31], selective activation of ERβ in cells may serve to suppress growth of estrogen-dependent cells. In our research (Fig. 5), *Qing E formula plus Danshen* enhanced luciferase activity of ERs in transiently transfected Hela cells. None of the *Qing E formula plus Danshen* was more potent than E_2_ in ERα, but the mixture at the concentration of 100 μg/ml showed noticeably more potent than E_2_ in ERβ. Meanwhile, the increase of estrogenic activities was obviously inhibited by ICI 182,780, an estrogen receptor antagonist. These results demonstrated that *Qing E formula plus Danshen* possesses significant estrogen-like activity with ERβ and ERα agonist activity, and a slightly higher affinity for ERβ in HeLa cells. This is in agreement with a previous study report that traditional Chinese herb contains compounds that could be considered to be potential selective estrogen receptor modulators (SERMs) with specific agonist estrogenic activity [32, 33].

Postmenopausal osteoporosis is one of the major types of osteoporosis in humans. Animal models for postmenopausal osteoporosis are generated by ovariectomy. Mornitoring of BMD is important for diagnosis and the treatment of osteoporosis as decreased bone mass is a major characteristic of this disease. In this study, decreased BMD in OVX rats, determined by pQCT, was observed only in the metaphysic of tibia, trabecular bone, which contains osteoblasts and osteoclast on its surface and is more active in bone turnover and bone remodeling compared to cortical bone [34, 35]. Indeed, our analysis of OVX rats showed only loss of trabecular BMD.at the epiphyseal line down to 3.0 mm, but it was very significantly improved after 12-week oral administration of *Qing E formula plus Danshen* (Figs. 3 and 4). In other words, the density of cancellous bone proximal epiphysis line was improved significantly. This finding is consistent with previous studies that the loss of bone in adult OVX rats was more prominent in trabecular than cortical bone [36], and phytoestrogens have been implicated in the prevention of bone loss in postmenopausal osteoporosis [37]. The integrity of skeletal is maintained through a bone remodeling process that balances bone formation and bone resorption [38]. Bone loss occurs in estrogen deficiency due to enhanced bone resorption and impaired osteoblast function. phytoestrogen provides a protective effect against OVX-induced bone loss that is associated with decreased bone turnover through suppressing bone resorption. ERα induces osteoclast apoptosis, but the mechanism for impaired osteoblast function remains to be clarified [39]. The improvement in BMD following *Qing E formula plus Danshen* treatment may be partly attributed to its estrogenic activity (Fig. 5), and evidenced by increased uterine weight in *Qing E formula plus Danshen* exposed animals (Table 1), which is consistent with the report that *Danshen* is supporting bone healing [25].

Sex hormones strongly influence body fat distribution [40]. Ovariectomy-induced obesity has been attributed to metabolic changes as a result of ovarian hormone deficiency, which leads to increased fat synthesis and deposition in the adipocytes. When adipocytes reach their capacity of fat storage, fat becomes mobilized to be deposited in the viscera as the skeletal muscles, heart and liver (ectopic fat syndrome) [41]. That is frequently associated cardiovascular risk factors (dyslipidemia, atherosclerosis, and coronary artery disease). Our data showed a significant increase in the body weight of ovariectomised rats compared with the sham, although their food consumption was comparable throughout the study (Table 1). The increased body fat was either due to increased lipogenesis or decreased lipolysis or both [42]. In the present research, the ovariectomised rats showed significant increases in their TG and TC levels, particularly in the LDL fraction (Table 4). After 6-week oral administration of all 3 formulas to the OVX rats, LDL-C and TG levels were significantly reduced, but HDL-C/TC level was remarkably increased compared with the OVX rats. This indicated that all the *Qing E formula*s have evident beneficial effects on improving serum lipids to potentially reduce the risk of coronary heart disease with a dose-dependent effect and synergistic effect with *Danshen*. This is consistent with the report that phytoestrogens may contribute to the decreased incidence of postmenopausal cardiovascular disease [43].

All the data demonstrated that *Danshen* has the notable synergistic effect on promoting the estrogenic activities of *Qing E formula*. This verified the rationality of TCM cognition in menopausal pathogenesis: kidney deficiency and blood stasis. According to TCM, the effect of *Duzhong* and *Buguzhi* is to tonify the kidney, *Danshen* is to improve the blood circulation. Therefore, *Qing E formula plus Danshen* hits the pathogenesis, and it is more effective in treating menopausal disorders holistically. This is also why TCM aims toward healing rather than symptomatic treatment.

Although the results are encouraging, This study was the most preliminary research for *Qing E formula plus Danshen*. we had some shortness, eg. the medicinal mechanism was not very clear. So we still have a long way to go. In the following research, we will increase the quantity of rat samples, verify whether the effects of *Qing E formula plus Danshen* on bone mineral densities are adjusted by ERs, and investigate how the formula affects the metabolism of osteoblasts and osteoclast, to elucidate the underlying mechanisms more clearly.

## Conclusion

*Qing E formula plus Danshen* exerted more evident estrogenic effects than other groups in improving the structure of estrogen target organs such as uterus and bone, and modulating endocrine metabolism. These effects may be attributed to an alteration in gene expression of ERs modulated by the formula. Thus, *Qing E formula plus Danshen* has demonstrated a potential therapeutic use in the treatment of menopausal disorders.

## Abbreviations

BDD, *Qing E formula plus Danshen*; ERT, estrogen-replacement therapy; ERα, estrogen receptor alpha; ERβ, estrogen receptor beta; FSH, follicle-stimulating hormone; H&E, hematoxylin-eosin; HDL-C, high density lipoprotein cholesterol; HRT, Hormone-replacement therapy; LDL-C, low density lipoprotein cholesterol; LH, luteinizing hormone; OVX, ovariectomized; pQCT, peripheral quantitative computed tomography; SERMs, selective estrogen receptor modulators; TC, total cholesterol; TCM, Traditional Chinese Medicine; TG, triglycerides; TJUTCM, Tianjin University of Traditional Chinese Medicine.
